# Composite outcome of oral azvudine vs. nirmatrelvir-ritonavir in COVID-19 patients: a retrospective cohort study

**DOI:** 10.3389/fphar.2025.1546787

**Published:** 2025-04-04

**Authors:** Jingxia Chen, Zhengyue Liu, Ruolin Liu, Chengxin Su, Yunyun Yang, Zhuo Wang

**Affiliations:** Department of Pharmacy, Shanghai Changhai Hospital, The First Affiliated Hospital of Navy Medical University, Shanghai, China

**Keywords:** COVID-19, azvudine, nirmatrelvir-ritonavir, real-world study, composite outcome

## Abstract

**Objective:**

To explore the effectiveness and safety of azvudine and nirmatrelvir-ritonavir in a real-world setting.

**Methods:**

This retrospective cohort study included adult patients with confirmed COVID-19 who received azvudine or nirmatrelvir-ritonavir treatment at Shanghai Changhai Hospital between 1 November 2022, and 30 March 2023. Data were collected from the hospital’s electronic medical record system using a standardized data extraction form. Propensity score matching (PSM) was used to control for potential confounding factors. The primary outcome was the incidence of composite disease progression, defined as the occurrence of death, ICU admission, invasive respiratory support, or high-flow oxygen therapy. Multivariable Cox regression analysis was performed to identify the factors independently associated with the composite progression outcomes.

**Results:**

This study included 476 patients: 296 treated with azvudine and 180 treated with nirmatrelvir-ritonavir. After PSM, 139 patients were included in each group. There were no statistically significant differences between the two groups regarding the composite outcome (log-rank: P = 0.475; HR: 0.82, 95%CI: 0.46–1.43, P = 0.478), death (log-rank: P = 0.526; HR: 0.82, 95%CI: 0.44–1.52, P = 0.528), ICU admission (log-rank: P = 0.525; HR: 0.69, 95%CI: 0.22–2.18, P = 0.526), invasive ventilation (log-rank: P = 0.814; HR: 1.20, 95%CI: 0.27–5.39, P = 0.814), or oxygen use (log-rank: P = 0.370; HR: 1.44, 95%CI: 0.65–3.18, P = 0.372). The multivariable analysis showed that the antiviral drug (HR = 0.861, 95%CI: 0.486–1.524, P = 0.607) was not independently associated with the composite outcome. Only severe COVID-19 was independently associated with the composite outcome (HR = 3.322, 95%CI: 1.569–7.031, P = 0.002). The safety outcomes were similar between the two groups.

**Conclusion:**

This real-world study demonstrates comparable efficacy and safety profiles between azvudine and nirmatrelvir-ritonavir in treating COVID-19 patients, regardless of disease severity or baseline characteristics. The findings support azvudine as a practical alternative for treatment selection, particularly in resource-constrained settings or for patients with contraindications to specific therapies. Clinical decisions should prioritize patient-specific needs, accessibility, and cost-effectiveness. Further large-scale prospective studies are needed to validate these observations and refine subgroup-specific treatment strategies.

## Introduction

COVID-19 is an infectious respiratory disease caused by SARS-CoV-2 and has imposed a huge burden on the socio-economic and medical systems around the globe (Koelle et al., 2022; Rothan and Byrareddy, 2020), with reported 775,251,779 cases and 7,043,660 deaths since early 2020 (World Health Organization, 2024). In February 2024, there were still 358 new severe cases and 22 deaths reported in China, indicating that COVID-19 continues to be a healthcare burden despite vaccination efforts (Lam et al., 2024; Schrimpf et al., 2023). Presently, the main strain prevalent in the world and in China is JN.1 (World Health Organization, 2024), which is highly infectious (Rubin, 2024). Currently, antiviral treatments such as nirmatrelvir/ritonavir (Paxlovid™) and azvudine (FNC) are the primary means of treating COVID-19 in China. Nirmatrelvir is a SARS-CoV-2 main protease (Mpro) inhibitor that prevents viral replication by blocking the cleavage of polyproteins, while ritonavir serves as a pharmacokinetic enhancer by inhibiting nirmatrelvir’s metabolism. Azvudine, a nucleoside analog, acts as an RNA-dependent RNA polymerase inhibitor that terminates viral RNA chain elongation, thereby inhibiting viral replication. Antiviral drugs can prevent disease progression or patient death by preventing virus replication, significantly reducing hospitalization and mortality rates among COVID-19 patients and shortening the time to turn negative (Zhang et al., 2021; Uraki et al., 2022; Li et al., 2022).

Indeed, a phase III study on nirmatrelvir-ritonavir showed that among COVID-19 patients over 65 years old, the rates of hospitalization and death due to COVID-19 were significantly lower among those receiving the antiviral treatment compared with those who did not (Arbel et al., 2022). A randomized controlled trial demonstrated that compared with traditional antiviral therapy, azvudine shortened the time for nucleic acid conversion to negative among patients with mild to moderate COVID-19 (Ren et al., 2020). The benefits are also seen in the real world, and azvudine significantly reduces the incidence of composite disease progression outcomes compared with supportive treatment among hospitalized COVID-19 patients (Sun et al., 2023). On the other hand, studies directly comparing nirmatrelvir-ritonavir vs. azvudine are rare, and additional research is necessary.

Unfortunately, the available studies comparing azvudine vs. nirmatrelvir-ritonavir report conflicting results. Indeed, some studies report favorable outcomes with (Gao et al., 2023; Zhao et al., 2023), while others favor azvudine (Han et al., 2024; Deng et al., 2023; Wei et al., 2023) or report no differences between the two drugs (Chen et al., 2024; Wang S. et al., 2024). Therefore, there is still no conclusive evidence regarding the real-world head-to-head comparison of the treatment effects of the two drugs, and further research is still needed to explore the efficacy of azvudine and nirmatrelvir-ritonavir in the treatment of COVID-19.

Therefore, the present study aimed to explore the therapeutic effects and safety of azvudine and nirmatrelvir-ritonavir in a real-world setting. The results could help define the value of the two drugs in managing COVID-19.

## Methods

### Study design and patients

This retrospective cohort study was conducted at Shanghai Changhai Hospital, a tertiary care hospital in China. The target population included all adult COVID-19 patients who received antiviral therapy during the study period. A consecutive sampling method was employed to enroll eligible patients treated with azvudine or nirmatrelvir-ritonavir between 1 November 2022, and 30 March 2023. Propensity score matching (PSM) was applied to mitigate selection bias and potential confounding. Follow-up period was defined as the interval between hospital admission and discharge. All patients included in the final analysis had complete follow-up data throughout their entire hospitalization. The study was approved by the ethics review committee of Shanghai Changhai Hospital (#CHEC-2023-100). The requirement for individual informed consent was waived by the committee because of the retrospective nature of the study.

**FIGURE 1:**
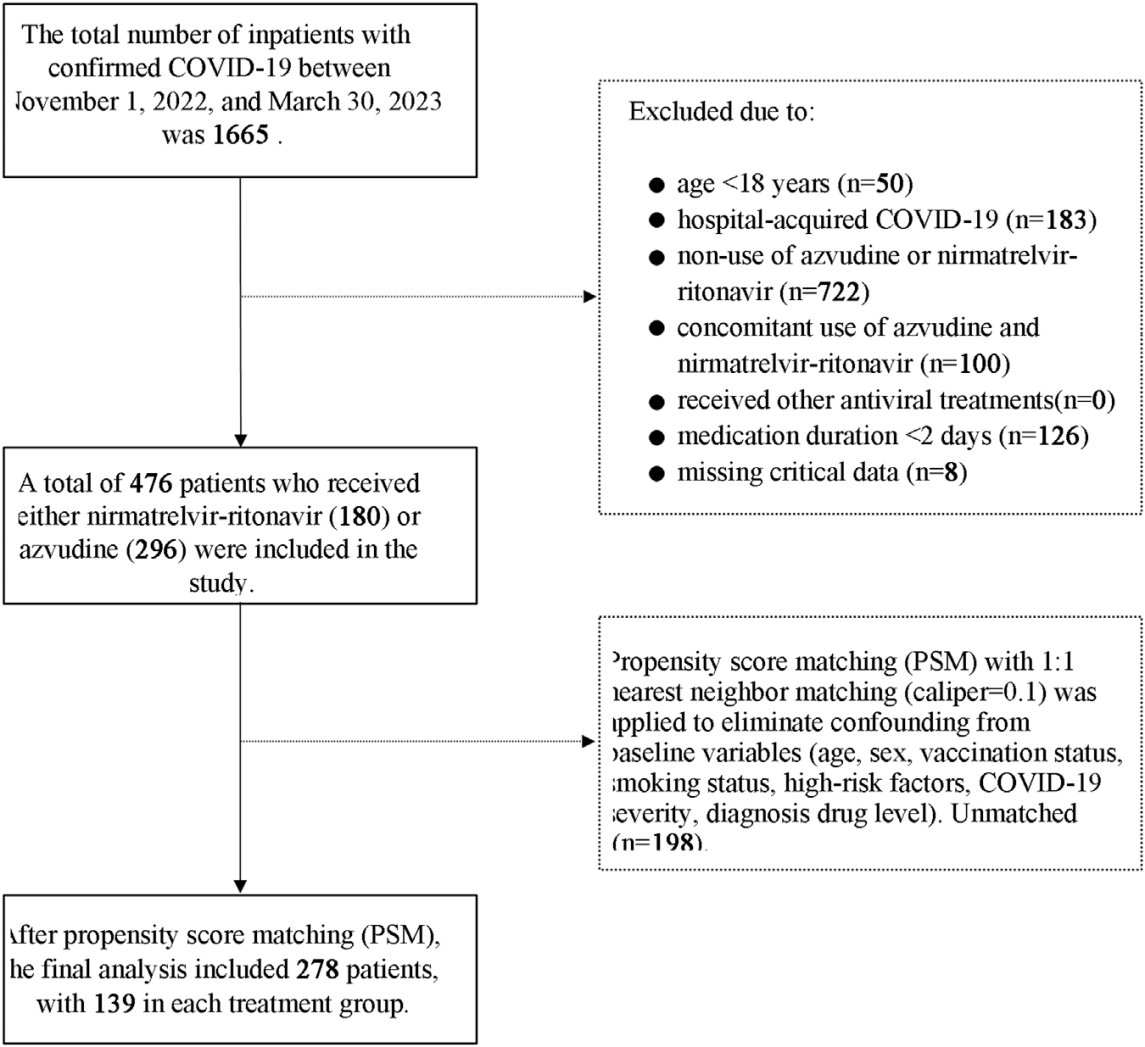
Patient selection and exclusion flow Diagram.

The inclusion criteria were 1) age ≥18 years, 2) diagnosed with COVID-19 infection according to the “Diagnosis and Treatment Protocol for COVID-19 Pneumonia” (i.e., RT-PCR Ct value < 35), and 3) received azvudine or nirmatrelvir-ritonavir. The exclusion criteria were 1) the antiviral drug was used for <2 days, 2) received other antiviral treatments, 3) concurrent use of azvudine and nirmatrelvir-ritonavir, 4) patients diagnosed with COVID-19 after hospitalization, or 5) missing key clinical information (Figure 1).

### Antiviral treatment

The antiviral treatment plan was selected by the patients and physicians after a comprehensive discussion based on the clinical features, physician’s experience, and according to the “Diagnosis and Treatment Protocol for COVID-19 Pneumonia”. The oral administration plan for nirmatrelvir-ritonavir was 300 mg nirmatrelvir +100 mg ritonavir (twice a day) for 5 days. The oral administration plan for azvudine was 5 mg once daily for 7 days or longer (not exceeding 14 days). The dosage of azvudine could be adjusted based on renal function if necessary. Other supportive treatments such as assisted ventilation, glucocorticoid therapy, non-steroidal anti-inflammatory therapy, nutritional support, anticoagulant therapy, etc., were applied routinely and according to the “Diagnosis and Treatment Protocol for COVID-19 Pneumonia”.

### Data collection

Patient demographic information and clinical data were collected from the medical record system of Shanghai Changhai Hospital, including age, sex, body mass index (BMI), vaccination status, smoking and drinking history, comorbid high-risk factors (including cardiovascular and cerebrovascular diseases [CVD, including hypertension], chronic lung diseases, type 2 diabetes mellitus [T2DM], chronic liver and kidney diseases, cancer, and immune deficiency), time from diagnosis to medication use, severity of COVID-19, concomitant medication use, history of comorbidities (including CVD, T2DM, liver disease, kidney disease, immune system diseases, schizophrenia, and cancer), and laboratory test results.

### Outcomes

The primary outcome was the incidence of composite disease progression, defined as the occurrence of death, intensive care unit (ICU) admission, invasive respiratory support, or high-flow oxygen therapy, whichever occurred first. The secondary outcome measures included the incidence of individual composite outcome components, length of hospital stay, time to negative nucleic acid conversion (from medication start to negativity), changes in Ct values, and cumulative occurrence of abnormal laboratory indicators.

### Statistical analysis

To mitigate potential confounding effects from clinical baseline variables (age, sex, vaccination status, smoking status, presence of high-risk factors, COVID-19 severity, and Diagnosis drug level) that may influence therapeutic outcomes, propensity score matching was employed. The caliper value was set at 0.1. The logistic regression was used to calculate the closest neighbor distance for 1:1 matching. The standardized mean difference (SMD) was used to assess the balance of the baseline variables between the two groups before and after propensity score matching, with an SMD of less than 0.1 indicating a good balance between the two groups.

In addition to propensity score matching, we employed inverse probability of treatment weighting (IPTW) as a sensitivity analysis to utilize the full sample and validate the robustness of our findings. The same set of covariates used in the propensity score model was included in the IPTW model. Balance after IPTW was evaluated using SMD, with values less than 0.1 indicating adequate balance.

The statistical analyses were performed using R 4.3.2 (The R Project for Statistical Computing, www.r-project.org). The continuous data were presented as means ± standard deviations or medians (ranges) and tested between groups using Student’s t-test. The categorical data were presented as n (%) and analyzed using the chi-squared test or Fisher’s exact test. Kaplan-Meier (KM) cumulative incidence curves were plotted for the incidence of the composite disease progression outcome or individual endpoints, and the log-rank test was used to compare the differences between groups. Hazard ratios (HRs) with corresponding 95% confidence intervals (CIs) were calculated using univariate Cox proportional hazards regression models. The association between age and survival was examined using restricted cubic spline (RCS) regression. The optimal number of knots was determined based on the Akaike Information Criterion (AIC) minimization principle. To identify factors independently associated with composite progression outcomes, multivariate Cox regression analysis was conducted on the matched dataset. Additionally, subgroup analyses were performed to evaluate the impact of baseline variables on therapeutic efficacy, with results visualized through forest plots. Two-sided P-values <0.05 were considered statistically significant.

## Results

### Characteristics of the patients

This retrospective cohort study enrolled 476 hospitalized COVID-19 patients, comprising 296 azvudine recipients and 180 nirmatrelvir-ritonavir recipients. Following 1:1 propensity score matching (PSM), 139 matched pairs were established with all standardized mean differences below 0.1, as detailed in Table 1. Subsequent inverse probability treatment weighting (IPTW) analysis demonstrated adequate balance across all baseline variables (standardized differences <0.1), although minor residual imbalances persisted in age (p = 0.036) and smoking status (p = 0.094). Comprehensive baseline characteristics for both analytic approaches are presented in Table 1.

**TABLE 1:** Characteristics of the patients.

	Before matching				After matching				IPTW result	
	Azvudine (n = 296)	Nirmatrelvir-ritonavir (n = 180)	P	SMD	Azvudine (n = 139)	Nirmatrelvir-ritonavir (n = 139)	P	SMD	t/χ^2^	P
Age	74.33 ± 14.82	76.89 ± 16.31	0.079	0.16	77.02 ± 13.90	78.99 ± 14.31	0.244	0.14	0.73	0.036
Sex (%)			0.146	0.15			0.895	0.03	0.76	0.030
Female	106 (35.81)	52 (28.89)			39 (28.10)	41 (29.50)				
Male	190 (64.20)	128 (71.10)			100 (71.90)	98 (70.50)				
Vaccine			0.214	0.17			0.991	0.02	1.00	0.009
Non-vaccinated	73 (24.70)	57 (31.70)			46 (33.10)	45 (32.40)				
Unknown	168 (56.80)	96 (53.30)			74 (53.20)	75 (54.00)				
Vaccinated	55 (18.60)	27 (15.00)			19 (13.70)	19 (13.70)				
Smoking			0.009	0.31			0.874	0.06	0.67	0.094
No	215 (72.60)	147 (81.70)			121 (87.10)	122 (87.80)				
Unknown	38 (12.80)	8 (4.40)			4 (2.90)	5 (3.60)				
Yes	43 (14.50)	25 (13.90)			14 (10.10)	12 (8.60)				
High-risk			0.333	0.10			0.858	0.04	0.90	0.013
No	40 (13.51)	31 (17.22)			17 (12.20)	19 (13.70)				
Yes	256 (86.50)	149 (82.80)			122 (87.80)	120 (86.30)				
Diagnosis drug level			0.200	0.17			>0.999	<0.001	0.99	0.015
≤5	90 (30.40)	42 (23.30)			28 (20.10)	28 (20.10)				
>5	201 (67.90)	133 (73.90)			110 (79.10)	110 (79.10)				
Unknown	5 (1.70)	5 (2.80)			1 (0.70)	1 (0.70)				
COVID-19 type			0.180	0.21			NaN	0.05	1.00	0.009
Unknown	3 (1.00)	2 (1.10)			0 (0)	0 (0)				
Mild	33 (11.10)	15 (8.30)			9 (6.50)	10 (7.20)				
Severe	118 (39.90)	90 (50.00)			67 (48.20)	69 (49.60)				
Moderate	142 (48.00)	73 (40.60)			63 (45.30)	60 (43.20)				

Categorical variables are shown as n (%).

Continuous variables are presented as mean ± standard deviation.

SMD: standardized mean difference.

Statistical tests under “PTW, result” column: t-test was used for continuous variables (age) and Chi-squared test (χ^2^) was used for categorical variables.

### Therapeutic outcomes

**FIGURE 2:**
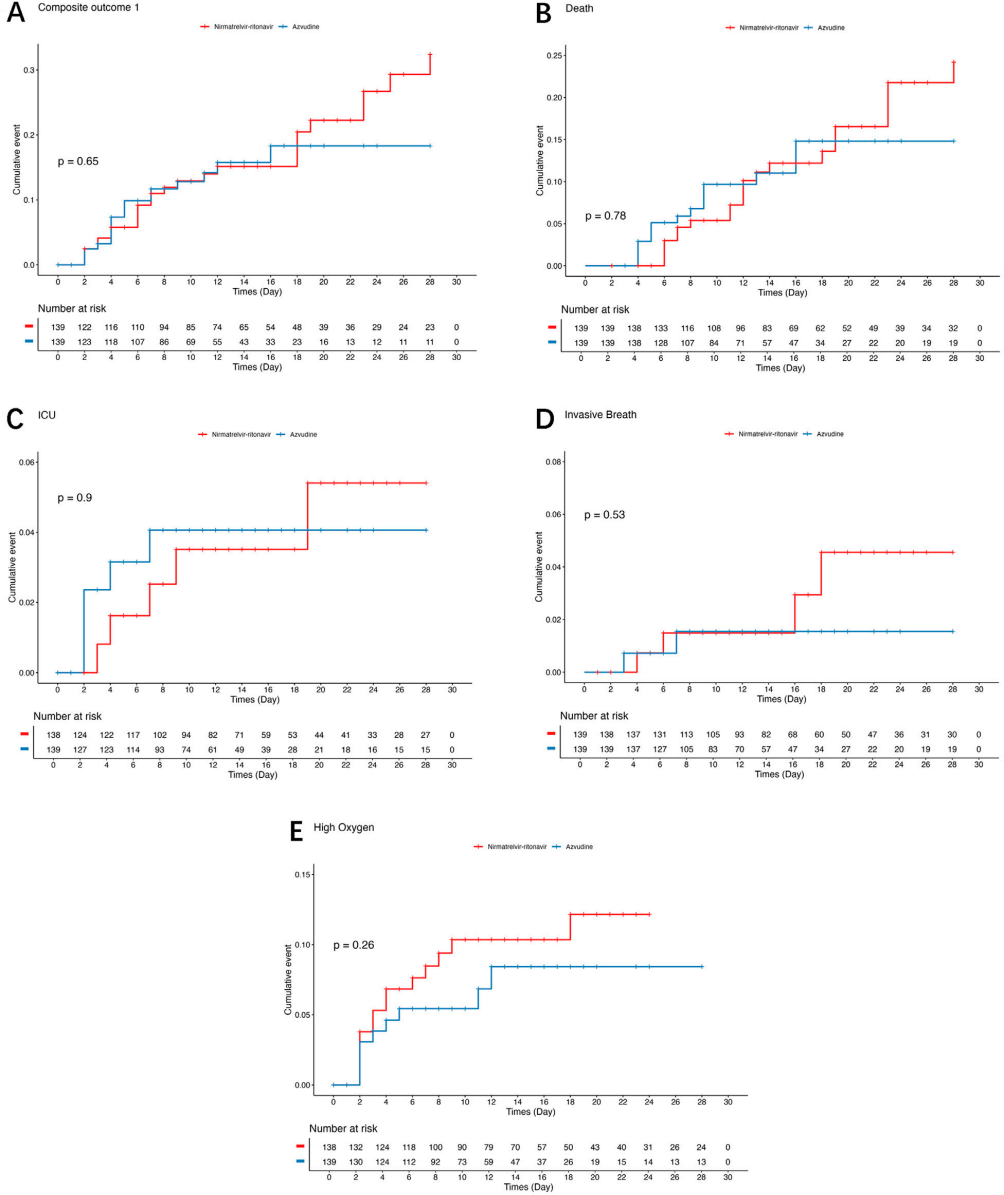
Kaplan-Meier curves for composite disease progression and individual outcomes. **(A)** Composite outcome of disease progression. **(B)** Death. **(C)** Invasive ventilation. **(D)** Intensive care unit admission. **(E)** Oxygen use.

As shown in Figure 2 and Table 2, there were no statistically significant differences between the azvudine and nirmatrelvir-ritonavir groups regarding the composite outcome (log-rank: P = 0.650; HR: 0.87, 95%CI: 0.47–1.60, P = 0.652), death (log-rank: P = 0.779; HR: 0.91, 95%CI: 0.47–1.77, P = 0.780), ICU admission (log-rank: P = 0.903; HR: 1.08, 95%CI: 0.31–3.75, P = 0.904), invasive ventilation (log-rank: P = 0.523; HR: 0.58, 95%CI: 0.11–3.20, P = 0.534), or oxygen use (log-rank: P = 0.265; HR: 0.63, 95%CI: 0.28–1.44, P = 0.272).

**TABLE 2:** Therapeutic outcomes of azvudine vs. nirmatrelvir-ritonavir.

Outcomes	Azvudine (n = 139)	Nirmatrelvir-ritonavir (n = 139)	P (log-rank)	HR (95% CI)	P	IPTW result		
						P (log-rank)	HR (95% CI)	P
Composite outcome*	18 (12.90%)	25 (18.00%)	0.650	0.87 (0.47,1.60)	0.652	0.670	1.04 (0.64,1.68)	0.873
Death	15 (10.80%)	21 (15.10%)	0.779	0.91 (0.47, 1.77)	0.780	0.460	1.42 (0.83, 2.43)	0.197
ICU	5 (3.60%)	5 (3.60%)	0.903	1.08 (0.31,3.75)	0.904	0.560	1.45 (0.55,3.84)	0.454
Invasive breath	2 (1.40%)	4 (2.90%)	0.523	0.58 (0.11,3.20)	0.534	0.850	1.23 (0.35,4.34)	0.746
High oxygen	9 (6.50%)	16 (11.50%)	0.265	0.63 (0.28, 1.44)	0.272	0.050	0.63 (0.33, 1.22)	0.174

*Reference = nirmatrelvir-ritonavir.

HR, hazard ratio; CI, confidence interval.

The relationship between age and survival outcomes was assessed using RCS regression. Based on the AIC minimization principle, models with 4 knots demonstrated optimal fit. Both unadjusted and covariate-adjusted RCS analyses revealed a predominantly linear association between age and survival (P for non-linearity = 0.319 and 0.083, respectively), as illustrated in Supplementary Figures S2. All covariates were modeled as follows: age as a continuous variable (in years), sex as a categorical variable (male/female), vaccination status as a categorical variable (vaccinated/non-vaccinated/unknown), smoking status as a categorical variable (yes/no/unknown), high-risk factors as a binary variable (yes/no), COVID-19 severity as a categorical variable (mild/moderate/severe), and diagnosis drug level as a categorical variable (<5, >5, unknown).

The multivariable Cox analysis showed that the antiviral drug (HR = 0.79, 95%CI: 0.42–1.49, P = 0.465) was not independently associated with the composite outcome (Table 3).

**TABLE 3:** Multivariable Cox regression analysis of the composite outcome.

Variables	HR	95%CI	P	IPTW result		
				HR	95%CI	P
Treatment						
Nirmatrelvir-ritonavir	Ref.			Ref.		
Azvudine	0.79	(0.42,1.49)	0.465	1.05	1.05 (0.65–1.68)	0.848
Age	0.99	(0.97,1.01)	0.359	1.00	1.00 (0.98–1.02)	0.922
Sex						
Female	Ref.			Ref.		
Male	1.86	(0.86,4.02)	0.115	1.23	1.23 (0.71–2.13)	0.466
Vaccine						
Non-vaccinated	Ref.			Ref.		
Vaccinated	1.10	(0.44,2.79)	0.839	0.76	0.76 (0.43–1.34)	0.340
Unknown	0.90	(0.43,1.87)	0.781	0.99	0.99 (0.48–2.04)	0.978
Smoking						
No	Ref.			Ref.		
Yes	0.41	(0.10,1.77)	0.234	0.59	0.59 (0.26–1.33)	0.204
Unknown	0.69	(0.09,5.34)	0.720	1.02	1.02 (0.40–2.60)	0.971
High-risk						
No	Ref.			Ref.		
Yes	2.48	(0.58,10.57)	0.219	5.30	5.30 (1.51–18.61)	0.009
Diagnosis drug level						
<5	Ref.			Ref.		
>5	1.06	(0.44,2.55)	0.903	1.00	1.00 (0.57–1.77)	1.000
unknown	15.48	(1.63,146.67)	0.017	7.09	7.09 (2.81–17.86)	0.000
COVID type						
Mild	Ref.			Ref.		
Moderate	0.77	(0.16,3.67)	0.741	1.96	1.96 (0.51–7.54)	0.327
Severe	2.97	(0.67,13.07)	0.150	5.92	5.92 (1.61–21.72)	0.007
Unknown	NA	NA (NA,NA)	NA	NA	NA	NA

CI, confidence interval.

### Subgroup analysis

**FIGURE 3:**
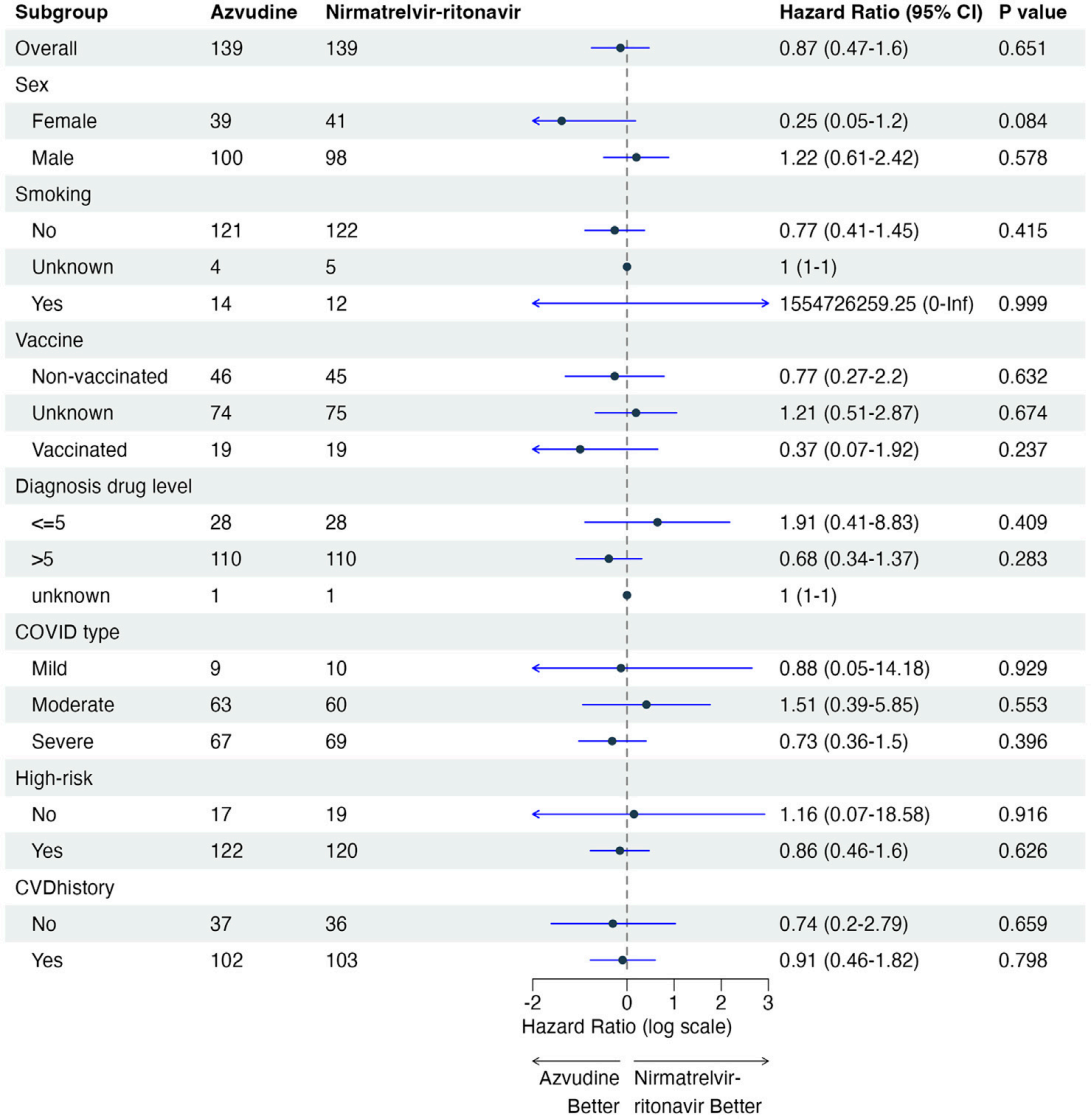
Subgroup analysis of the composite outcome of disease progression.

The subgroup analysis showed no statistically significant differences across all subgroups based on sex, smoking, vaccination, drug use at diagnosis, COVID-19 type (Figure 3).

### Changes in Ct values

**FIGURE 4:**
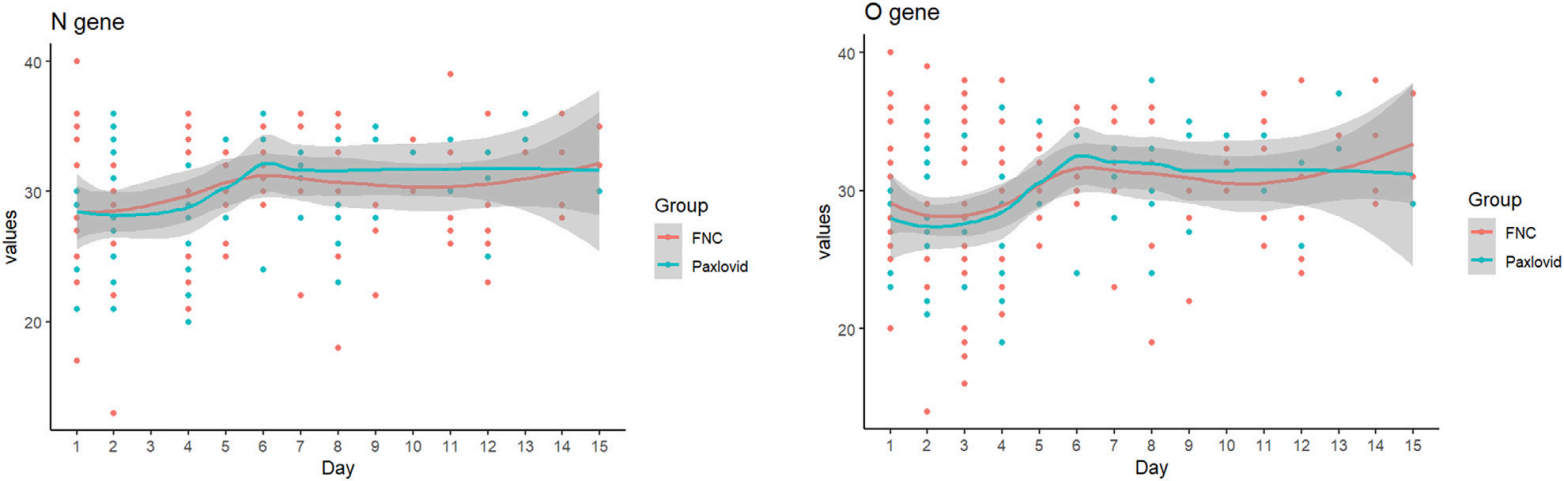
Changes in RT-PCR Ct values.

As shown in Figure 4, the patterns of changes in the Ct values were similar between the two groups, with a little dip in the Ct value of the O gene after 2–3 days of treatment.

### Safety

**FIGURE 5:**
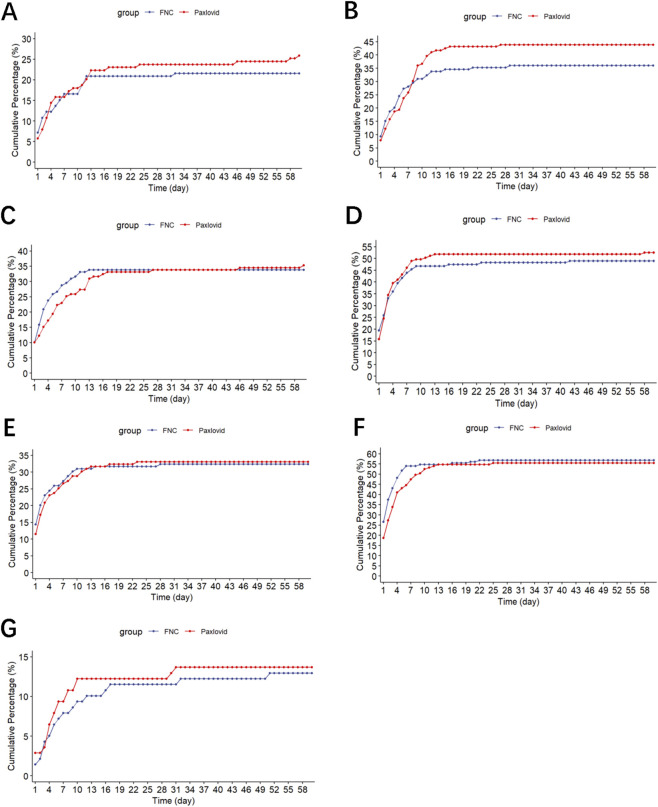
Cumulative Incidence of abnormal laboratory test values. **(A)** Alkaline phosphatase (ALP). **(B)** Alanine transaminase. **(C)** Aspartate transaminase **(D)** Blood urea nitrogen. **(E)** Creatinine. **(F)** Lactate dehydrogenase. **(G)** Total bilirubin.

The cumulative incidence of laboratory abnormalities within 60 days was similar between the two groups (azvudine vs. nirmatrelvir-ritonavir) for alkaline phosphatase (ALP) (21.6% vs. 25.9%) (Figure 5A), alanine aminotransferase (ALT) (36.0% vs. 43.9%) (Figure 5B), aspartate aminotransferase (AST) (33.8% vs. 35.3%) (Figure 5C), blood urea nitrogen (BUN) (48.9% vs. 52.5%) (Figure 5D), creatinine (CRE) (32.4% vs. 33.1%) (Figure 5E), lactate dehydrogenase (LDH) (56.8% vs. 55.4%) (Figure 5F), and total bilirubin (TBIL) (12.9% vs. 13.7%) (Figure 5G). No other adverse events were reported in either group.

## Discussion

This study explored the therapeutic effects and safety of azvudine and nirmatrelvir-ritonavir in a real-world setting. The results suggest that azvudine exhibits similar real-world therapeutic effects and safety compared with nirmatrelvir-ritonavir. There were no differences between the two drugs in terms of the composite outcome that included death, ICU admission, invasive ventilation, and oxygen use or the individual outcomes.

Randomized controlled trials demonstrated the efficacy and safety of azvudine (de Souza et al., 2023; Wang Y. et al., 2024) or nirmatrelvir-ritonavir (Liu et al., 2023; Amani and Amani, 2023) for managing COVID-19. Still, clinical trials are performed in highly selected patients, limiting the generalizability of the conclusions. Real-world studies are complementary to clinical trials (Kim et al., 2018). Furthermore, no clinical trials directly compared azvudine vs. nirmatrelvir-ritonavir. Real-world studies can provide some evidence pending a clinical trial.

Gao et al. (2023) reported a retrospective study that showed that compared with patients receiving azvudine, those receiving nirmatrelvir-ritonavir showed faster virus suppression and earlier nucleic acid negative conversion during the initial hospitalization stage (Gao et al., 2023). It is similar to another recent retrospective study that showed that in terms of conversion time, azvudine and nirmatrelvir-ritonavir had similar efficacy, while nirmatrelvir-ritonavir was able to suppress the virus faster in patients with mild COVID-19 (Zhao et al., 2023). Nirmatrelvir-ritonavir might have an advantage in reducing early mortality compared with azvudine (Han et al., 2024). On the other hand, other retrospective studies found that compared with nirmatrelvir-ritonavir, azvudine improved the composite disease progression outcomes among hospitalized COVID-19 patients (Deng et al., 2023; Wei et al., 2023). Of note, Deng et al. (Deng et al., 2023) observed a significant difference for the composite outcome (P = 0.026) but not for the individual outcomes (all P > 0.05). Chen et al. (Chen et al., 2024) reported that azvudine was comparable to nirmatrelvir/ritonavir and molnupiravir in adult patients with mild-to-moderate COVID-19 in terms of nucleic acid negative conversion, hospital stay, and adverse events. Similar results were reported in older adults with severe COVID-19 (Wang S. et al., 2024). The study found no differences between the two drugs in the composite outcome of disease progression or the individual outcomes, which contradicts previous studies but agrees with others (Chen et al., 2024; Wang S. et al., 2024). Therefore, further exploration is needed to compare the head-to-head efficacy of these two drugs. Based on the available evidence, one or the other can be used to manage patients with COVID-19. The choice should be made based on drug availability and the financial capacities of the patients.

In the present study, the choice of antiviral was not independently associated with the composite outcome. Only COVID-19 severity was independently associated with the composite outcome, which is unsurprising considering that it is the most important prognostic indicator in patients with COVID-19 (Wiersinga et al., 2020; Tan et al., 2021). Furthermore, the performance of the two drugs was similar across the various subgroups. Azvudine appears to be safe in patients on hemodialysis, suggesting that it can be used safely in patients with chronic kidney disease (Shang et al., 2023). On the other hand, nirmatrelvir-ritonavir is contraindicated in patients with severe or end-stage kidney disease, although the literature suggests it might be safe (Cai et al., 2023). Additional studies are necessary to determine the categories of patients that are the best fit for a specific treatment.

In this study, abnormalities in ALP (21.6% vs. 25.9%), ALT (36.0% vs. 43.9%), AST (33.8% vs. 35.3%), BUN (48.9% vs. 52.5%), CRE (32.4% vs. 33.1%), LDH (56.8% vs. 55.4%), and TBIL (12.9% vs. 13.7%) were observed. Of note, it was impossible to determine whether a given abnormality in a given patient was due to the antiviral drug, COVID-19, or comorbidities. Nevertheless, those abnormalities were observed in previous studies of azvudine and nirmatrelvir-ritonavir (Zhang et al., 2021; Ren et al., 2020; Sun et al., 2023; Gao et al., 2023; Zhao et al., 2023; Han et al., 2024; Deng et al., 2023; Wei et al., 2023; Chen et al., 2024; Wang et al., 2024a; de Souza et al., 2023; Wang Y. et al., 2024; Liu et al., 2023; Amani and Amani, 2023; Shang et al., 2023; Cai et al., 2023), and no new safety signals were observed.

A rebound phenomenon has been described for the Ct value after starting nirmatrelvir-ritonavir or molnupiravir (Parums, 2022; Anderson et al., 2022; Charness et al., 2022; Rubin, 2022), while it was suggested that such a rebound was not observed with azvudine (da Silva et al., 2023). Although the present study showed a modest dip in the O-gene Ct values at the beginning of treatment with the two drugs, no evident rebound was observed.

A strength of this study was the inclusion of all patients with COVID-19 who were hospitalized at the study hospital during the study period. In China, hospitalization was mandatory during the COVID-19 pandemic whenever an RT-PCR test was positive, resulting in the inclusion of all patients. Nevertheless, this study had limitations. The patients were from a single hospital, resulting in a small sample size. Subgroup analyses were conducted post-matching without subgroup-specific adjustments, and the results should be interpreted cautiously as exploratory due to limited sample sizes within certain subgroups, which may reduce statistical power. We did conduct an IPTW analysis, and the conclusions were consistent with our previous findings. Our empirical assessments of non-linearity confirmed that modeling age as a continuous linear term in our Cox proportional hazards models and propensity score calculations was appropriate for our dataset. This approach allowed us to maximize statistical efficiency while accurately representing the relationship between age and the outcomes of interest. In addition, although the “Diagnosis and Treatment Protocol for COVID-19 Pneumonia” was applied to all patients, there is a risk of bias due to local practice, limiting generalizability. The study was retrospective, limiting the data to those available in the patient charts. Larger prospective studies and randomized controlled trials are needed in the future.

In conclusion, azvudine exhibits similar real-world therapeutic effects and safety compared with nirmatrelvir-ritonavir in patients with COVID-19, irrespective of patient characteristics or COVID-19 severity. These findings have important clinical implications for treatment selection, particularly in resource-limited settings or for patients with contraindications to either medication. Our results suggest that clinicians can consider both drugs as viable treatment options, with the choice guided by patient-specific factors, drug availability, and cost considerations. Future prospective studies with larger sample sizes are warranted to confirm these findings and potentially identify specific patient subgroups that might benefit more from one treatment over the other.

## Data Availability

The datasets presented in this article are not readily available because It’s patient privacy. We can’t provide any information. Requests to access the datasets should be directed to chenjingxia_2012@163.com.
