# Supplementary material for: Composite outcome of oral azvudine vs. nirmatrelvir-ritonavir in COVID-19 patients: a retrospective cohort study
Source: Front Pharmacol. 2025 Apr 4;16:1546787. doi: 10.3389/fphar.2025.1546787 (PMC12006181; doi:10.3389/fphar.2025.1546787)
Supplement: Supplementary file 1 [file DataSheet1.pdf]

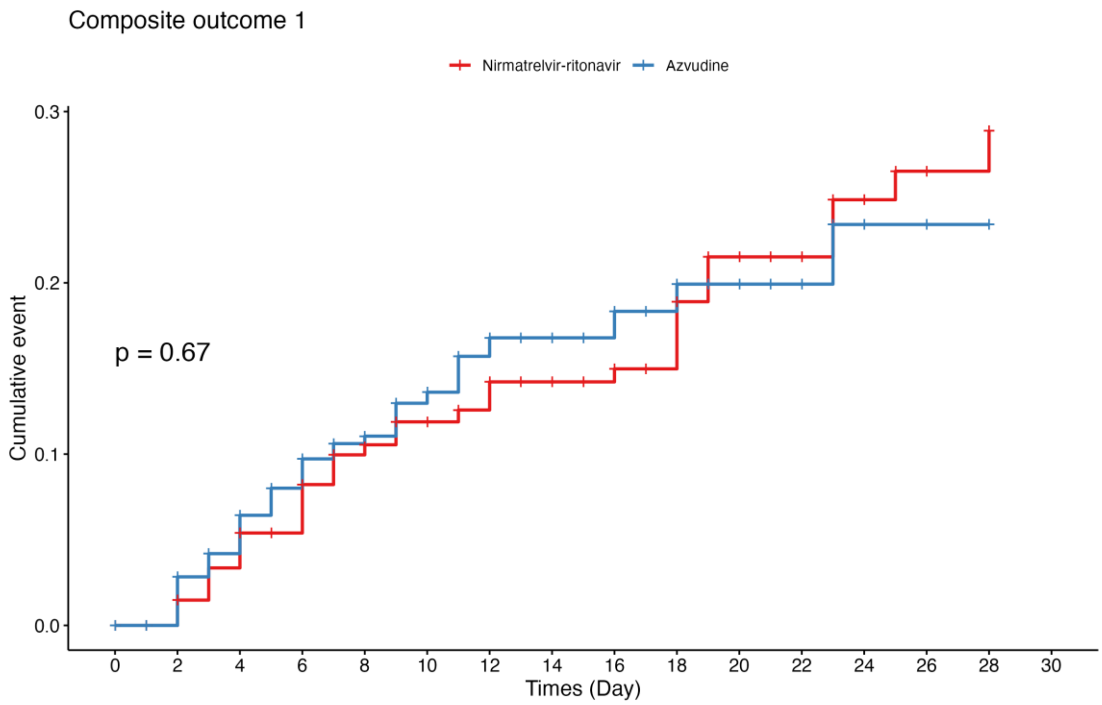

Figure S 1.1 Composite outcome survival_plot(IPTW)

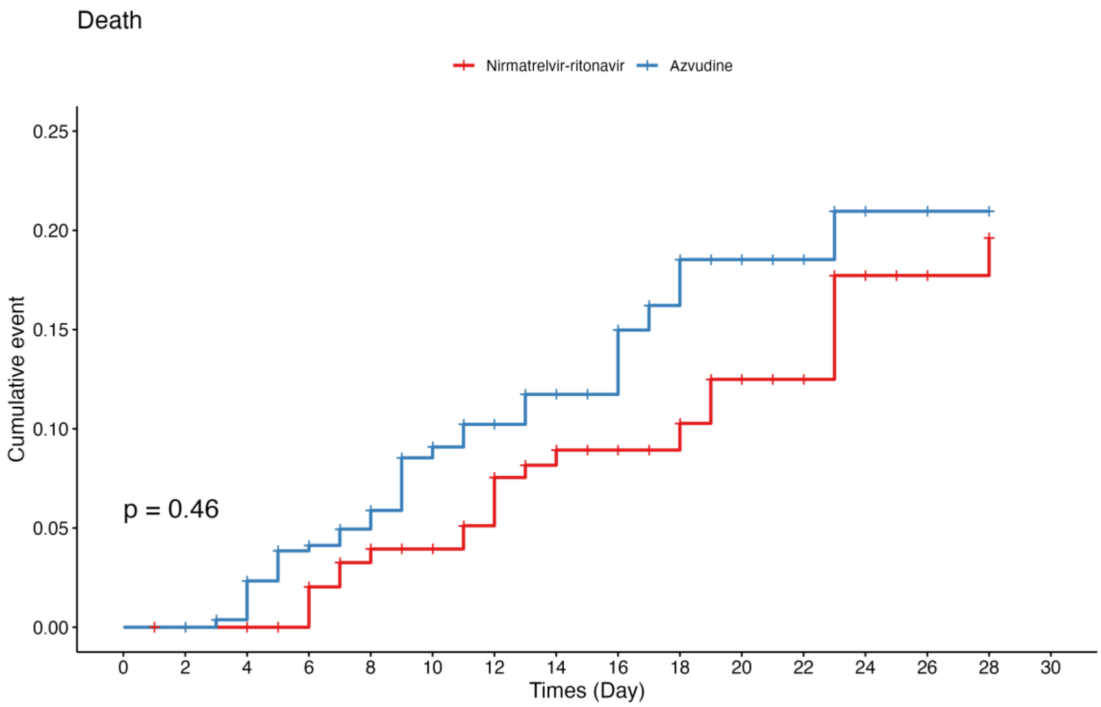

Figure S 1.2 Death survival_plot(IPTW)

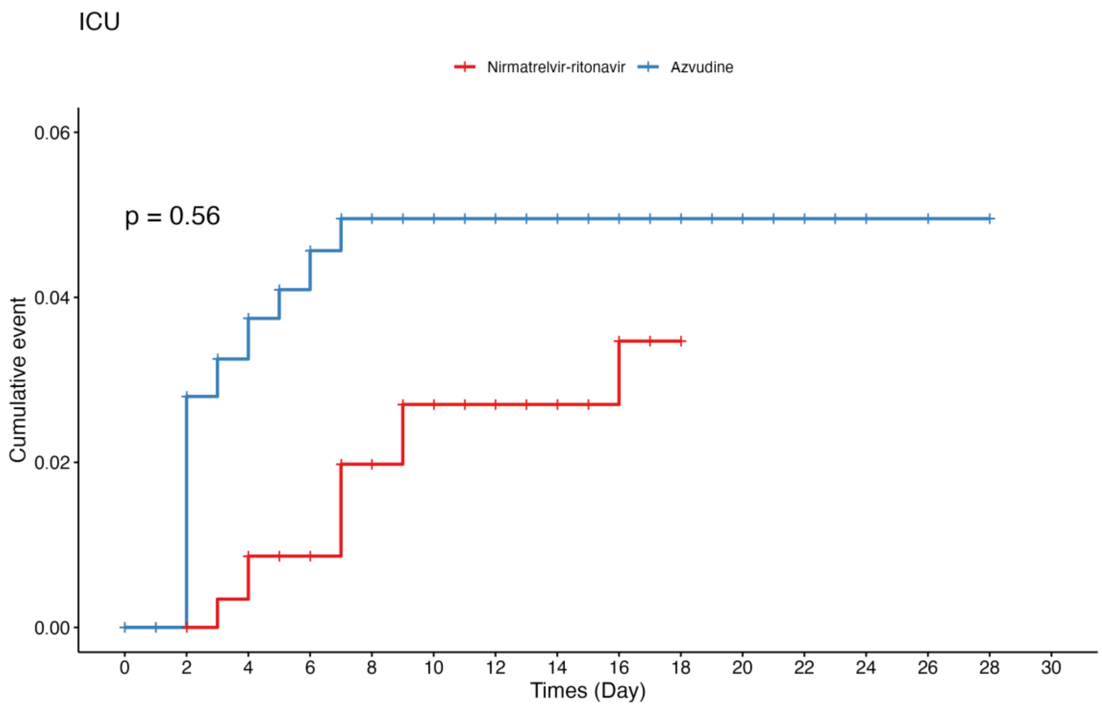

Figure S 1.3 ICU survival_plot(IPTW)

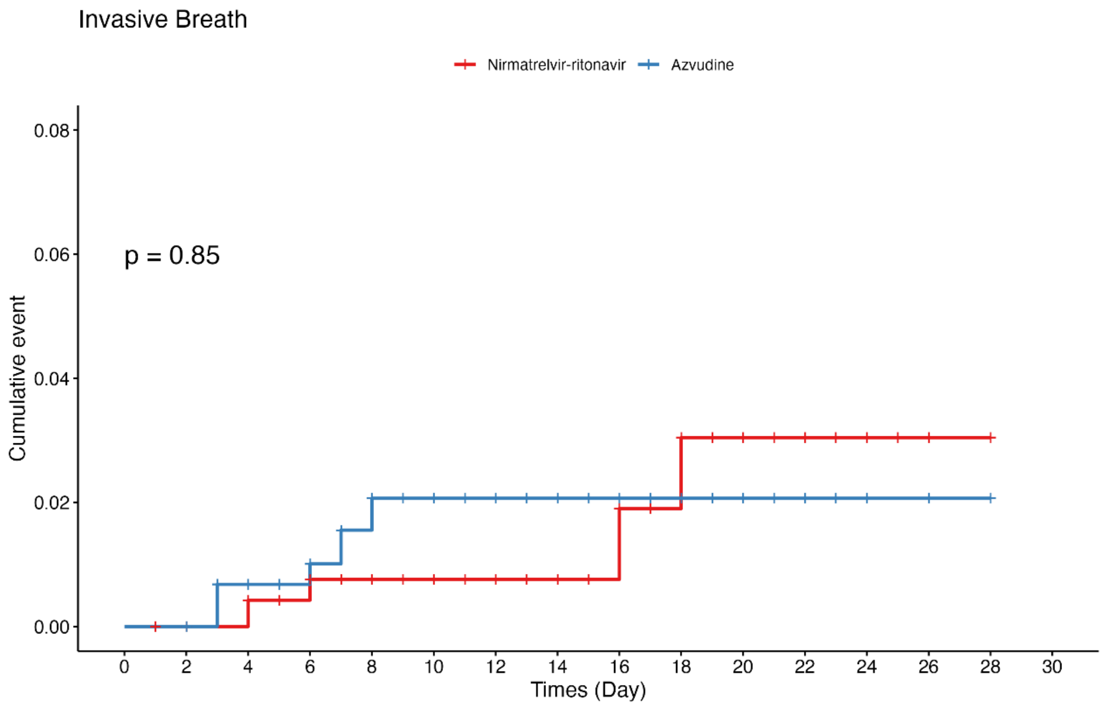

Figure S 1.4 Invasive Breath survival_plot(IPTW).

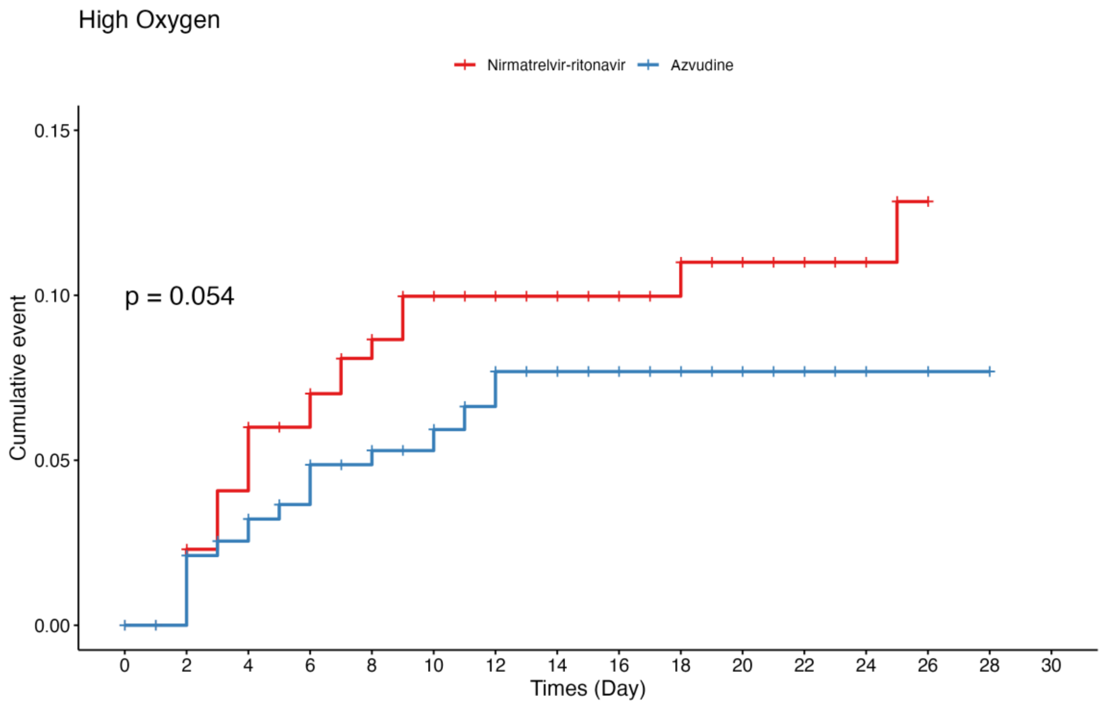

Figure S 1.5 High Oxygen survival_plot(IPTW)

Table S1 Characteristics of the patients(IPTW)

		**Before IPTW**				**After IPTW**			
		**Paxlovid**	**FNC**	**p**	**SMD**	**Paxlovid**	**FNC**	**p**	**SMD**
Age (mean (SD))		76.89 (16.31)	74.33 (14.82)	0.079	0.164	74.48 (17.72)	75.07 (14.50)	0.731	0.036
Sex (%)	Female	52 (28.89)	106 (35.81)	0.146	0.148	147.75 (31.50)	156.98 (32.90)	0.76	0.03
	Male	128 (71.11)	190 (64.19)			321.27 (68.50)	320.08 (67.10)		
Vaccine (%)	Non-vaccinated	57 (31.67)	73 (24.66)	0.214	0.165	131.53 (28.04)	132.24 (27.72)	0.996	0.009
	Unknown	96 (53.33)	168 (56.76)			256.30 (54.65)	262.83 (55.09)		
	Vaccinated	27 (15.00)	55 (18.58)			81.19 (17.31)	81.99 (17.19)		
Smoking (%)	No	147 (81.67)	215 (72.64)	0.009	0.308	366.00 (78.03)	363.16 (76.12)	0.667	0.094
	Unknown	8 (4.44)	38 (12.84)			32.29 (6.88)	45.13 (9.46)		
	Yes	25 (13.89)	43 (14.53)			70.74 (15.08)	68.77 (14.41)		
High-risk (%)	No	31 (17.22)	40 (13.51)	0.333	0.103	74.72 (15.93)	73.75 (15.46)	0.895	0.013
	Yes	149 (82.78)	256 (86.49)			394.30 (84.07)	403.31 (84.54)		
Diagnosis drug level (%)	<=5	42 (23.33)	90 (30.41)	0.2	0.171	126.98 (27.07)	132.14 (27.70)	0.988	0.015
	>5	133 (73.89)	201 (67.91)			332.11 (70.81)	335.29 (70.28)		
	unknown	5 (2.78)	5 (1.69)			9.93 (2.12)	9.64 (2.02)		
COVID type (%)	Mild	15 (8.33)	33 (11.15)	0.18	0.21	47.18 (10.06)	48.42 (10.15)	1	0.009
	Moderate	73 (40.56)	142 (47.97)			208.89 (44.54)	214.12 (44.88)		
	Severe	90 (50.00)	118 (39.86)			208.26 (44.40)	209.62 (43.94)		
	Unknown	2 (1.11)	3 (1.01)			4.69 (1.00)	4.89 (1.03)		

Table S2 Therapeutic outcomes of azvudine vs. nirmatrelvir-ritonavir(IPTW)

	P (log-rank)	HR (95% CI)
Composite outcome	0.67	1.04 (0.64,1.68) 0.873
Death	0.46	1.42(0.83, 2.43) 0.197
ICU	0.56	1.45(0.55,3.84) 0.454
Invasive breath	0.85	1.23 (0.35,4.34) 0.746
High oxygen	0.05	0.63 (0.33, 1.22) 0.174

Table S3 Results of Multivariate Cox Regression Analysis (IPTW)

	HR	95%CI	P
Treatment			
Nirmatrelvir-ritonavir	Ref.		
Azvudine	1.05	1.05(0.65-1.68)	0.848
Age	1.00	1.00(0.98-1.02)	0.922
Sex			
Female	Ref.		
Male	1.23	1.23(0.71-2.13)	0.466
Vaccine			
Non-vaccinated	Ref.		
Unknown	0.76	0.76(0.43-1.34)	0.340
Vaccinated	0.99	0.99(0.48-2.04)	0.978
Smoking			
No	Ref.		
Yes	0.59	0.59(0.26-1.33)	0.204
Unknown	1.02	1.02(0.40-2.60)	0.971
High-risk			
No	Ref.		
Yes	5.30	5.30(1.51-18.61)	0.009
Diagnosis drug level			
<5	Ref.		
>5	1.00	1.00(0.57-1.77)	1.000
unknown	7.09	7.09(2.81-17.86)	0.000
COVID type			
Mild	Ref.		
Moderate	1.96	1.96(0.51-7.54)	0.327
Severe	5.92	5.92(1.61-21.72)	0.007
Unknown	0.00	0.00(0.00-0.00)	0.000

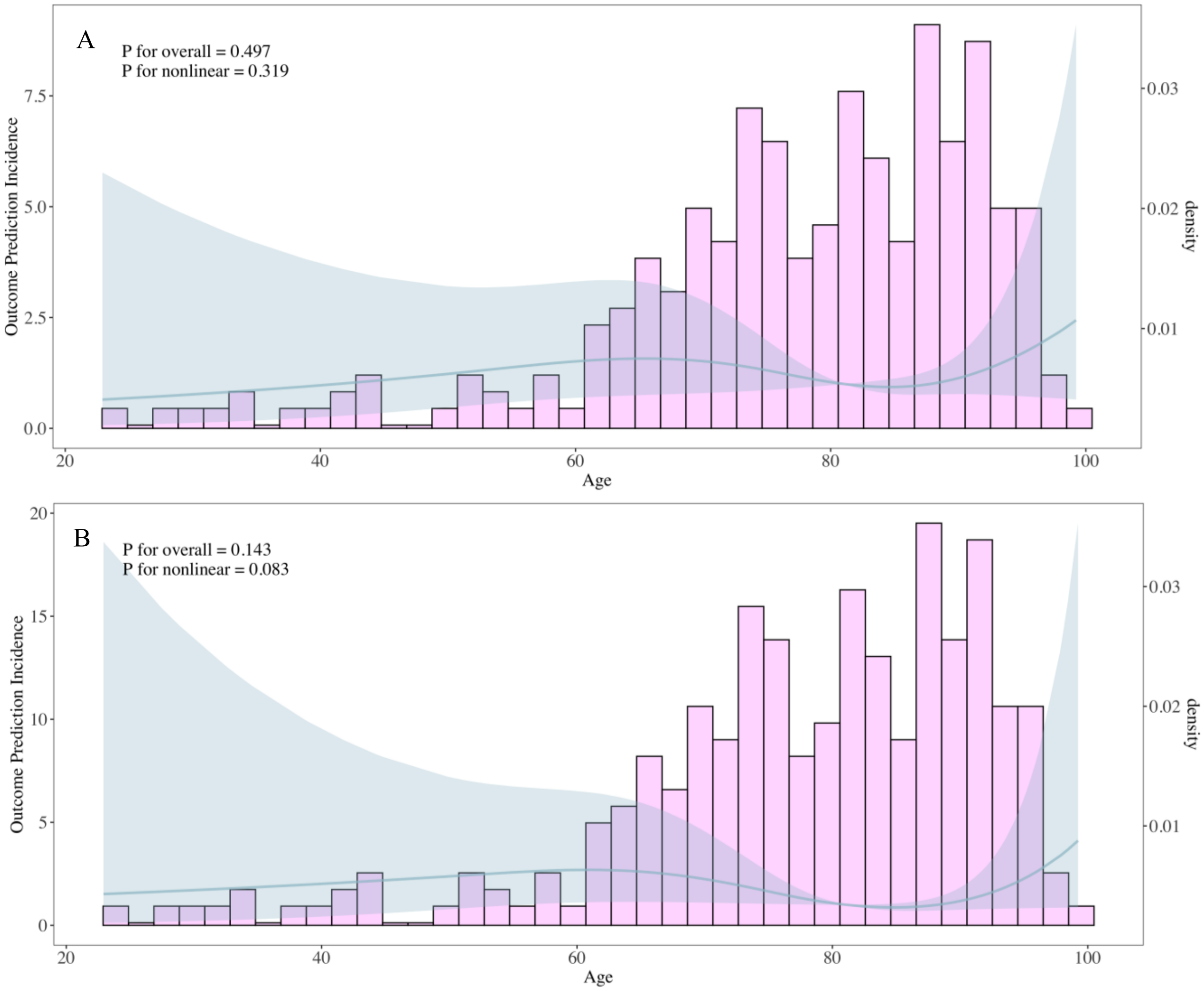

Figure S2 RCS analysis results for age and survival (A: unadjusted covariates; B: adjusted covariates).
